# Clinical implications of metastatic lymph node ratio in gastric cancer

**DOI:** 10.1186/1471-2407-7-200

**Published:** 2007-10-24

**Authors:** Caigang Liu, Ping Lu, Yang Lu, Huimian Xu, Shubao Wang, Junqing Chen

**Affiliations:** 1 Department of Oncology, First Affiliated Hospital of China Medial University, Shenyang, China

## Abstract

**Background:**

The 5-year survival rate in patients with gastric cancer is still poor, and lymph node metastasis is considered one of the most important prognostic factors. However, there are controversies in the classification of lymph node metastasis in gastric cancer. This study was carried out to investigate whether the metastatic lymph node ratio is a reliable classification of lymph node metastasis in gastric cancer in Chinese.

**Methods:**

224 cases with gastric cancer with more than D1 dissection were retrospectively reviewed. The association between the total number of resected lymph nodes and the number of metastatic lymph nodes was determined. The prognostic value of the metastastic node ratio, defined as the ratio of the number of metastatic lymph nodes over the total number of resected lymph nodes, and the pN classification was assessed.

**Results:**

The number of metastatic lymph node increased with the number of total resected lymph nodes. A Cox regression revealed that the metastatic node ratio, the number of metastatic nodes, histological type, and histological growth pattern independently influenced prognosis. The 5-year survival rates were 78%, 61%, 25%, 0% in cases with a metastastic node ratio of 0%, > 0% but < 40%, 40–80%, > 80%, respectively (*P *< 0.001), and were 78%, 62%, 38%, 0% in cases with gastric cancer histologically classified as pN0, pN1, pN2, pN3, respectively (*P *< 0.001).

**Conclusion:**

The metastatic lymph node ratio is a simple and useful independent prognostic factor. It may obviate possible confounding factors that are related to stage migration, and should be considered as an important component in the lymph node category.

## Background

Gastric cancer remains a major cause of cancer death, and the 5-year survival rate in patients with gastric cancer is still poor despite improved survival due to early detection, rational lymphadenectomy and several therapeutic modalities [1]. Lymph node metastasis is considered one of the most important prognostic factors, and accurate categorization of lymph node metastasis or optimization of pN category is fundamentally critical for decision making of the subsequent therapies after surgery [2-4]. Thus, the rational categorization of resected lymph nodes will help further improve therapeutic efficacy [1].

However, there are controversies in the classification of lymph node metastasis in gastric cancer. In Japan, the classification of lymph node metastasis is based on the anatomical station of metastatic lymph nodes [5]. However, in Western countries, it is classified by the number of metastatic regional lymph nodes according to the tumor node metastasis (TNM) staging categories established by the International Union against Cancer (UICC) [6]. Previously, it has been shown that lymph node metastasis is a significant prognostic factor in gastric cancer [7,8]. Some previous studies have demonstrated that the total number of metastatic lymph nodes is a reliable indicator as a prognostic factor than anatomical lymphatic spread [9-12]. Moreover, a few recent studies have suggested that the metastatic lymph node ratio is a more reliable prognostic factor [13-17]. However, the clinical values of these pathological parameters have not been fully verified. In addition, most studies on the prognostic significance of the number and ratio of metastatic lymph nodes in gastric cancer were carried out in western countries, and relevant data are virtually lacking in China. Therefore, the aim of this retrospective study was to investigate whether the metastatic lymph node ratio is a reliable classification of lymph node metastasis in gastric cancer in Chinese.

## Methods

### Patients

425 cases with gastric cancer (36 early and 389 advanced) were treated at the Department of Oncology, First Affiliated Hospital of China Medical University, Shenyang, China, between 1997 and 2002.

The inclusion criteria for this study included: 1), patients who received curative resection; 2), patients who underwent a lymph node dissection beyond limited (D1) dissection, i.e. D1 dissection + dissection of lymph nodes along the left gastric artery, D1 dissection + dissection of lymph nodes along the common hepatic artery, D1 dissection + dissection of lymph nodes along the celiac artery, extended (D2) dissection, or superextended (D3) dissection; and 3), patients in whom more than 15 lymph nodes were resected and pathologically examined [18].

The exclusion criteria included 1), patients who received a palliative operation; 2), patients who underwent a D1 lymph node dissection; 3), patients who had metastatic lymph nodes in retropancreatic, mesenteric, duodenohepatic ligament, or para-aortic lymph node metastasis were excluded; and 4), patients with liver metastasis and peritoneal dissemination.

Based on the inclusion and exclusion criteria, 201 patients were excluded from the Study; 12 of the 36 cases with early gastric cancer and 98 cases with advanced gastric cancer received D1 lymph node dissection, and/or had less than 15 lymph nodes resected for pathologically examination. 34 patients had metastatic lymph nodes in retropancreatic, mesenteric, duodenohepatic ligament, or para-aortic lymph node metastasis. 57 cases (including those with liver metastasis and peritoneal dissemination) received a palliative operation. Therefore, a total of 224 patients with gastric cancer were included in the study. Their demographic and clinical characteristics are shown in Table 1.

**Table 1:** Demographic data and clinical and pathological characteristics of patients

Characteristics		Number of cases
Sex	Male	153
	Female	71
Age (years old)	≤ 60	114
	> 60	110
Tumor number	Single	208
	Multitude	16
Location of tumor	U (upper third stomach)	9
	M (middle third stomach)	41
	L (lower third stomach)	174
Maximum tumor diameter (cm)	≤ 2	28
	2–4	85
	> 4	111
pT category	pT1	34
	pT2	128
	pT3	59
	pT4	3
Histological type	G1 (well differentiated)	41
	G2 (moderately differentiated)	46
	G3 (poor differentiated)	137
Histological growth pattern		
	Expanding type	115
	Infiltrative type	109
Lymphatic vessel infiltrate	Present	59
	Absent	165
pN category	pN0	55
	pN1	87
	pN2	49
	pN3	33
Metastatic node ratio (%)	0	55
	1–19	72
	20–39	30
	40–59	26
	60–79	20
	80–100	21

The study protocol was approved by the Ethics Committee of China Medical University.

### Surgically dissection of lymph nodes

Lymph nodes were meticulously dissected from the enbloc specimens, and the classification of the dissected lymph nodes was determined by surgeons who reviewed the excised specimens after surgery based on the Japanese Classification of Gastric Carcinoma [5]. Then the resected lymph nodes were sectioned and stained with hematoxylin and eosin and examined for metastasis by pathologists. Clinical and histopathologic data of each patient were collected and recorded in a specifically designed data collection form. From the 224 cases, a total of 6316 lymph nodes (range 15–75 per patient) were picked up and histologically examined (Table 1).

### Classification of lymphadenectomy

Based on the Japanese Classification of Gastric Carcinoma (JCGC), lymph nodes were classified as Group 1 (the perigastric lymph nodes), Groups 2 (the lymph nodes along the left gastric artery, the common hepatic artery, and the splenic artery and around the celiac axis [5]. However, when the tumor is located in the lower third stomach, the lymph nodes along the splenic artery are classified as being in Group 3) and Group 3 (lymph nodes in the hepatoduodenal ligament, at the posterior aspect of the head of the pancreas, and at the root of the mesentery). Accordingly, lymphadenectomy was classified as D1, dissection of all the Group 1 lymph nodes; D2, dissection of all the Group 1 and Group 2 lymph nodes; and D3, dissection of all the Group 1, Group 2 and Group 3 lymph nodes.

pN category was defined as pN0 (no metastatic lymph node), pN1 (1–6 metastatic lymph nodes), pN2 (7–15 metastatic lymph nodes) and pN3 (> 15 metastatic lymph nodes), according to the 5^th ^Edition of UICC [18]. The metastatic lymph node ratio was calculated by dividing the total number of lymph nodes that have been removed and examined by the number of metastatic lymph nodes. The ratio was rated in six grades, from 0 and to 100%, with an increment of every 20% (Table 1).

The location of tumors was defined as upper, middle and lower third gastric cancer, according to JCGC and the histological type was defined as differentiated and undifferentiated, according to UICC [5,18]. In addition, histological growth patterns were also defined as expanding and infiltrative types [19].

### Statistical analysis

The correlation of the total number of dissected lymph nodes with pN catergory and the metastatic lymph node ratio was evaluated by curve fitting. We also examined the functional form of the covariate under study by Kaplan-Meier and Log rank test were adopted in the analysis of survival rate comparison. Univariate analysis and Martingale residual analysis were used to determine the association between the metastatic lymph node ratio and survival. Multivariate analysis was performed by using the Cox proportional hazards model selected in forward stepwise. All the data were analyzed with SPSS 13.0 statistics software (Chicago, IL United States). A *P *value of less than 0.05 was considered statistically significant.

## Results

### Correlation of pN catergory and the metastatic lymph node ratio with the total number of dissected lymph nodes

The total number of metastatic nodes was significantly influenced by the extension of the lymphadenectomy in gastric cancer (*F *= 29.085, *P *= 0.000).

**Figure 1:**
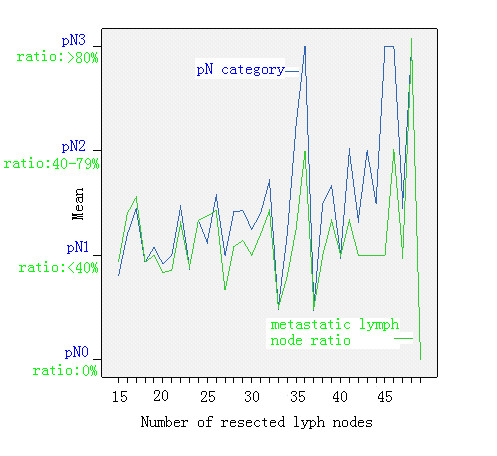
**The correlation of pN category and the metastatic lymph node ratio with the number of total resected lymph nodes**. The cuvres of pN and the metastatic nodes ratio ascended while the total number of dissected nodes increased, with the cuvre of pN category increased more significantly than metastatic node ratio, especially when the total number of the dissected nodes was more than 25.

The curve of pN category and the metastatic node ratio ascended while the number of total dissected nodes increased. In addition, the curve of pN category increased more significantly than metastatic node ratio category, especially when the total number of the dissected nodes was more than 25 (Figure 1).

### Survival

**Figure 2:**
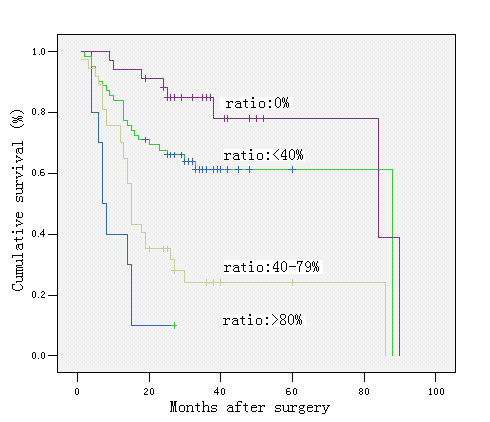
Survival curve and comparison of cumulative survival rates after surgery according to the metastatic lymph node ratio, calculated by dividing the total number of lymph nodes that have been removed and examined by the number of metastatic lymph nodes (0%, < 40%, 40–79, and ≥ 80%). There were significant differences among the groups (P < 0.00; Kaplan-Meier and log-rank test).

**Figure 3:**
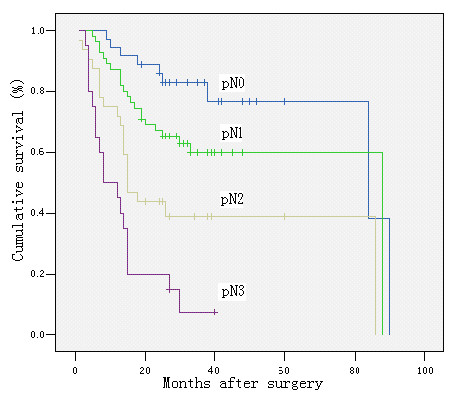
Survival curve and comparison of cumulative survival rates after surgery according to according to pN categories (pN0: no metastatic lymph node, pN1: 1–6 metastatic lymph nodes, pN2: 7–15 metastatic lymph nodes, and pN: > 15 metastatic lymph nodes). There were significant differences among the groups (P < 0.00; Kaplan-Meier and log-rank test).

**Figure 4:**
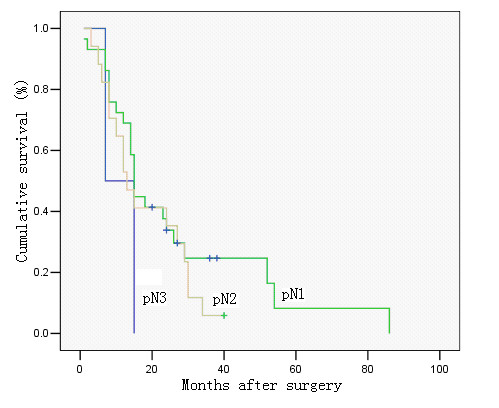
Survival curve of cases with metastatic lymph node ratio 40–79%, in relation to pN category. No significant difference was observed in cumulative survival rates after surgery among the groups (pN1, pN2 and pN3) (P = 0.367; Kaplan-Meier and log-rank test).

**Figure 5:**
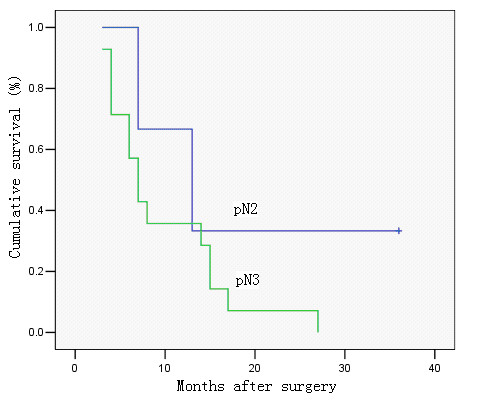
Survival curve of cases with metastatic lymph node ratio > 80%, in relation to pN category. No significant difference was observed in cumulative survival rates after surgery between the two groups (pN2 and pN3) (P = 0.224; Kaplan-Meier and log-rank test).

**Figure 6:**
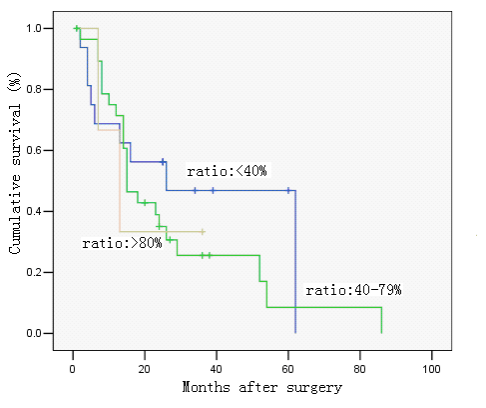
Survival curve of cases with pN2, in relation to metastatic lymph node ratio. No significant difference was observed in cumulative survival rates after surgery among the groups (< 40%, 40–79, and ≥ 80%) (P = 0.606; Kaplan-Meier and log-rank test).

**Figure 7:**
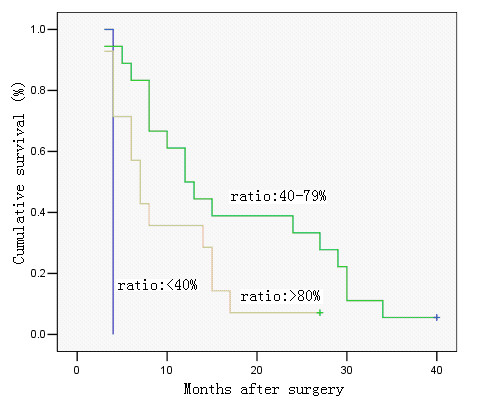
Survival curve of cases with pN3, in relation to metastatic lymph node ratio. There was a significant difference in cumulative survival rates after surgery among the groups (< 40%, 40–79, and ≥ 80%) (P = 0.025; Kaplan-Meier and log-rank test).

The 5-year survivals were 78%, 61%, 25%, 0% in cases with a metastastic node ratio of 0%, < 40%, 40–79, and ≥ 80%, respectively (*P *< 0.00), and 78%, 62%, 38%, 0% in cases with pN0, pN1, pN2, and pN3, respectively (*P *< 0.00). There was only a slight difference in the survival rates between patients with pN0 and pN1 and between those with a metastatic lymph node ratio of 0 and < 40%, but the survival rates decreased significantly in other groups (Figures 2 &3). Further analyses revealed that in patients with a metastatic node ratio of 40–79% and cases with the ratio of ≥ 80%, there was no significant difference in survival among the patients with pN1, pN2 and pN3 (Figures 4 &5). However, in cases with pN3, there was a significant difference in the survival rate among the patients with a lymph node ratio of < 40%, 40–79% and ≥ 80%) (*P *= 1/20.025) (Figure 6), although this difference was absent in patients with pN1 and pN2 (Figure 7).

### Metastatic lymph node ratio as a prognostic risk factor

The relative risk showed an increasing value from 1.866 to 12.554 as the metastatic lymph node ratio group increased (Table 2). Since the hazard ratios between categories 40–59% and 60–79%, and between 1–19% and 20–39% were very similar, the metastatic lymph node ratio was re-rated into four different grades (0, < 40%, 40–79 and ≥ 80%) (Table 2). The correlation between pN stages and the ratio grades is shown in Table 3.

**Table 2:** Univariate analysis of the metastatic lymph node ratio as a risk factor for survival

Metastatic lymph node ratio (%)		Hazard ratio	95% confidence interval	*P *value
Original grading	0			
	1–19	1.866	1.032–3.375	0.039
	20–39	1.916	1.060–3.463	0.031
	40–59	4.063	2.052–8.044	< 0.001
	60–79	5.101	2.636–9.870	< 0.001
	80–100	12.554	5.660–27.846	< 0.001
				
Revised grading	0			
	1–39	1.878	1.039–3.396	0.037
	40–79	4.574	2.546–8.217	< 0.001
	80–100	12.784	6.541–24.987	< 0.001

**Table 3:** Correlation between pN category and metastatic lymph node ratio

		Metastatic lymph node ratio (%)				Total number
		0	1–39	40–79	80–100	
pN category	pN0	55	0	0	0	55
	pN1	0	85	2	0	87
	pN2	0	16	30	3	49
	pN3	0	1	14	18	33

Total number		55	102	46	21	224

### Multivariate analysis of prognostic factors

The total number of metastatic lymph nodes (with pN category) and the metastatic lymph node ratio (with the 4-grade category) were evaluated along with other potential prognostic factors (including sex, age, the number of tumor, maximum tumor diameter, pT category, location of tumor, histological type, and histological growth pattern and lymphatic vessel infiltrate) for the prognostic significance in the multivariate analysis by using Cox regression (Table 4).

**Table 4:** Multivariate analysis of the metastatic lymph node ratios and the other clinico-pathological characteristics

Characteristics		Hazard ratio	95% confidence interval	*P *value
Sex (male vs. female)		0.731	0.469–1.138	0.166
Age (years old)		1.026	1.006–1.045	0.009
Tumor number		1.299	0.706–2.391	0.401
Location of tumor				
	Middle/Upper	2.612	0.601–11.361	0.200
	Lower/Upper	1.605	0.362–7.117	0.534
Maximum tumor diameter (cm)				
	2–4/≤ 2	3.841	1.317–11.208	0.014
	> 4/≤ 2	4.546	1.572–13.143	0.005
pT category				
	pT2/pT1	1.025	0.376–2.795	0.961
	pT3/pT1	2.687	0.979–7.325	0.055
	pT4/pT1	1.157	0.192–6.982	0.874
Histological type				
	G2/G1	2.163	1.086–4.308	0.028
	G3/G1	2.313	1.285–4.136	0.005
Histological growth pattern (expanding vs. infiltrative)		0.923	0.579–1.470	0.736
lymphatic vessel infiltrate (present vs. absent)		0.814	0.504–1.316	0.401
pN category				
	PN1/pN0	1.980	1.043–3.758	0.037
	pN2/pN0	2.987	1.138–7.841	0.026
	pN3/pN0	4.380	1.201–15.966	0.025
Metastatic lymph nodes ratio (%)				
	1–39/0	1.980	1.043–3.0785	0.037
	40–79/0	4.726	1.502–14.872	0.008
	80–100/0	11.841	2.787–50.321	0.001

The lymph node ratio category, the metastatic lymph node ratio, age, the maximum tumor diameter, histological type, were revealed to be independent prognostic factors, with the metastatic lymph node ratio being the most significantly independent facror (Table 4).

## Discussion

At present, the classification of metastatic lymph nodes in gastric cancer is still under extensive evaluation and investigation. In Japan, the JCGC classification that is based on the anatomical location of nodal involvement has been widely used [5]. However, some onco-surgical scholars in Western countries advocate that quantitative evaluation based on the number of metastatic lymph nodes is more predictive of patient survival than evaluation based on anatomical lymphatic spread [20,21]. Furthermore, there is no consensus on the number of lymph nodes to be dissected and examined for accurate staging of gastric cancer. In the western world, D1 (limited) lymph node dissection is generally performed, and thus it is difficult to have more than 15 nodes histologically examined for the cases. This problem has been identified by Mullaney et al. [22], who found that only 31% of cases with surgically resected gastric cancer could be accurately assessed according to the TNM system, suggesting the need of an improved of nodal staging. In Japan and some other Asian countries, D2 (extensive) lymph node dissection is a standard procedure for most cases, where more than 30 lymph nodes are routinely resected and histologically examined [23-26]. Therefore, the pN category may be not suitable for the cases from whom only a small number of nodes are resected and examined. Moreover, it is unclear whether the pN category could be influenced by the extension of lymphadenectomy in gastric cancer, and whether the metastatic lymph node ratio may truly prevent the phenomenon of stage migration, especially in Asian populations such as in Chinese.

In the present study, the correlation between the number of metastatic lymph nodes and the total number of dissected lymph nodes were determined. We found that the number of metastatic lymph nodes was influenced by the total number of dissected nodes. Moreover, we also observed that the pN category was influenced by the extension of lymphadenectomy more significantly than the metastatic node ratio. This observed phenomenon is in agreement with those reported by some other investigators, and may be explained by the following factors: 1), the number of picked up lymph nodes from the resected specimen varies among surgeons or pathologists expended different efforts [16,26]; 2) lymph nodes of large size or those macroscopically suspected to be metastatic were examined; and 3) the number of lymph nodes in gastric cancer varies in a great range in different patients, so the total number of examined nodes have influence on pN category [1,9,10]. However, the influence owing to the large range of total number could be reduced by the ratio. Thus, the metastatic node ratio would decrease, resulting in the induction of stage migration.

It has been previous suggested that the number of metastatic lymph nodes is a prognostic factor for gastric cancer [9-12]. Recent studies, most carried out in western populations, have demonstrated that metastatic lymph node ratio is a more reliable prognostic factor [12,14-17,27]. In this study of Chinese patients, we determined the survival rates in patients with gastric cancer, according to the pN catergory and metastatic lymph node ratio. We observed there was no significant deference in the survival rates between patients with pN0 and those with pN1 (pN category) and between patients with 0% and those with < 40% (the metastatic lymph node ratio). Moreover, we noticed that in patients with a metastatic node ratio of 40–79% and cases with the ratio of ≥ 80%, there was no significant difference in survival rate among the patients with pN1, pN2 and pN3. However, in cases with pN3, there was a significant difference in the survival rate among the patients with a lymph node ratio of < 40%, 40–79% and ≥ 80%, suggesting that the lymph node ratio is a better prognostic indicator than pN category, at least for cases with pN3.

Furthermore, we observed that hazard ratio in survival analysis was increased from 1.878 to 12.784 in patients with a metastatic lymph node ratio of 1–39%, 40–79% and ≥ 80%. Multivariate analysis further identified that the metastatic lymph node ratio was a most important independent prognostic factor amongst the other factors evaluated, including pN category. These findings emphasize the importance of metastatic lymph node ratio, as a reliable prognostic factor, to be included in a more accurate lymph node classification system.

## Conclusion

Lymph node ratio category has advantages in providing a more precise prognostic value than the pN category(5th edition, UICC). We recommend that classification of nodal status be established by a combination of both the metastatic nodes number and ratio, which would be the best category to provide both rational lymph node dissection and the foundation for adjunctive therapy and predict the prognosis [15,27-29].

## Competing interests

The author(s) declare that they have no competing interests.

## Authors' contributions

CGL and PL conceived the study, analysed data, and drafted the manuscript and submitted the manuscript. YL and HMX revised the manuscript critically for important intellectual content, and contributed to the date analysis. SBW and JQC conceived of the study and helped in drafting the manuscript. All authors read and approved the final manuscript.

## Pre-publication history

The pre-publication history for this paper can be accessed here:


